# Recent Trends in Rapid Environmental Monitoring of Pathogens and Toxicants: Potential of Nanoparticle-Based Biosensor and Applications

**DOI:** 10.1155/2015/510982

**Published:** 2015-03-25

**Authors:** Preeyaporn Koedrith, Thalisa Thasiphu, Jong-Il Weon, Rattana Boonprasert, Kooranee Tuitemwong, Pravate Tuitemwong

**Affiliations:** ^1^Faculty of Environment and Resource Studies, Mahidol University, Phutthamonthon District, Nakhon Pathom 73170, Thailand; ^2^Department of Safety Engineering, Institute of Environmental Medicine for Green Chemistry, Dongguk University, Gyeongju, Gyeongbuk 780-714, Republic of Korea; ^3^Department of Microbiology, Kasetsart University, Bangkok 10900, Thailand; ^4^Food Safety Center, Institute for Scientific and Technological Research and Services (ISTRS), King Mongkut's University of Technology Thonburi (KMUTT), Bangkok 10140, Thailand

## Abstract

Of global concern, environmental pollution adversely affects human health and socioeconomic development. The presence of environmental contaminants, especially bacterial, viral, and parasitic pathogens and their toxins as well as chemical substances, poses serious public health concerns. Nanoparticle-based biosensors are considered as potential tools for rapid, specific, and highly sensitive detection of the analyte of interest (both biotic and abiotic contaminants). In particular, there are several limitations of conventional detection methods for water-borne pathogens due to low concentrations and interference with various enzymatic inhibitors in the environmental samples. The increase of cells to detection levels requires long incubation time. This review describes current state of biosensor nanotechnology, the advantage over conventional detection methods, and the challenges due to testing of environmental samples. The major approach is to use nanoparticles as signal reporter to increase output rather than spending time to increase cell concentrations. Trends in future development of novel detection devices and their advantages over other environmental monitoring methodologies are also discussed.

## 1. Introduction

Environmental pollution is the major source of problem to human health and sustainable development of society and economy. The presence of environmental pathogens and their toxins, heavy metals, and organic pollutants is a serious environmental issue that drew scientific interest and public concern [1–3]. Several environmental microorganisms cause different clinical diseases and morbidities, resulting in public health burden. Particularly, the presence of water-borne pathogens in water streamline is critical issue for regulatory agencies, healthcare agencies, and industry sectors. These pathogens should therefore be routinely monitored under clinical diagnostic procedures. For instance,* Cryptosporidium parvum*, one of water-borne pathogens that contaminated drinking water, can cause disease even at low levels. The detection of water-borne pathogen using commercially available assays has been successfully achieved with detection limit of 10–1,000 organisms per mL (such as the widely used MERIFLUOR Cryptosporidium/Giardia test from Meridian Biosciences). Owing to low numbers of target microorganisms, step of culture preenrichment become necessary to increase their numbers to detectable level for several hours. Likewise, sensitive polymerase chain reaction- (PCR-) based techniques require preenrichment culturing process to efficiently detect environmental pathogens. In accordance with the US Environmental Protection Agency (EPA) Methods 1622 and 1623, preconcentration by filtration of 10-liter water sample is essential for detection of* C. parvum* oocysts.

The environmental sector urgently needs diagnostic system and test kits which are sensitive, cost-effective, and portable. Potential applications of nanotechnology enable researchers to use pathogen diagnostics as well as developing a new generation of biosensors and imaging techniques with higher sensitivity and reliability. Particularly, high sensitivity fluorescent dye doped nanoparticles provide important feature for increasing the signals by the magnitude of 10^5^ to 10^6^ times as well as tagging pathogens, enabling the instrument to detect specimens at very low levels. Furthermore, nanobiotechnology improves the performance of instrument for wider commercial deployment of the instrument at environmental site.

Nanoparticles (about 1–100 nm in diameter) display unique properties over bulk-sized materials and thus have been widely used in various areas, including biomedical, electronic, environmental, pharmaceutical, cosmetic, and energy [4, 5]. Indeed, environmental monitoring and diagnostics have been improved by using nanoparticles for detecting biotic and abiotic contaminants (e.g., pathogens and their toxins as well as metal ions and organic pollutants, resp.). Incorporating the nanoparticles into nanosensors provides advantages of rapid and high-throughput detecting ability on a portable device. The nanoparticles are considered as potential sensing materials due to strong physical confinement of electrons at nanoscale. Their tiny size correspond high surface-to-volume ratios. Additionally, their physical properties can be customized since they are directly related to size, composition, and shape. Surface-modified nanocolloids, such as gold nanoparticles (GNPs) [6], magnetic nanoparticles (MNPs) [7], quantum dots (QDs) [8], and carbon nanotubes, exhibit specific target-binding properties. Therefore, the distinct small size and nanoscale properties of nanoparticles are useful for new-generation environmental detection.

Use of fluorescence nanoparticles in combination with magnetic beads capable of capturing and concentrating target specimens in the sampling process has been established to overcome the limitation of fluorescence intensity [9–12]. Fluorescent nanoparticles (about 10–20 *μ*m diameter in range) including semiconductor nanoparticles, quantum dots, metal nanoparticles, silica nanoparticles, and polymer nanoparticles have been focused for research and development. Fluorescent nanoparticles display distinct features, such as high fluorescence intensity, photostability, photobleaching resistance, and biocompatibility. Their emission spectra show narrow peaks. The emission wavelength peak or fluorescent color can be adjusted based on their particle size during production. Based on the fact that one excitation source or wavelength with spectrum of ultraviolet or blue that excites certain fluorescent nanoparticles with different sizes simultaneously yields multiple emission spectra, multicolorimetric or multiplex assay format can be accomplished using a single light source [13]. The fluorescent nanoparticles are also served as multivalent scaffolds for supramolecular assemblies as well as versatile synthetic platform for surface coatings via chemical conjugation to bioreceptors (such as antibodies, aptamers, and other agents) [14]. Owing to their excellent properties, they become powerful tools for monitoring several different species under both biological and environmental purposes. For example, antibody- and DNA aptamer-based assays with and without the use of magnetic beads-mediated capture and filtration can detect bacteria as low as 10 cells per mL and several thousands of* C. parvum* oocysts [15–17]. In this review, the applications and developments of fluorescent nanoparticles and other potential nanoparticles are focused in terms of chemical and biological sensing within the environmental samples.

## 2. Potential Applications of Nanotechnology

Conventional molecular-based detection techniques are commonly used to identify pathogenic agents with high degree of sensitivity and reproducibility [18]. Mostly, these techniques cannot be employed in the field (e.g., rivers and drinking water distributors) since they usually require complex instrumentation and well-trained operator. Expensive and short shelve half-life of certain reagents (e.g., enzymes and oligonucleotides) also limits the utility of conventional pathogen detection techniques in rural areas of developing countries. Despite their high sensitivity, current technologies like enzyme-linked immunosorbent assay (ELISA) and polymerase chain reaction (PCR) still require extensive sample preparation and have long readout periods, resulting in delayed response and disease containment. Thus, taking advantage of the unique properties of nanoparticles (e.g., electrical, magnetic, luminescent, and catalytic capacity), economical detection assays with high speediness and sensitivity can be developed to promptly monitor environmental specimens, especially microbial pathogens. Nanoparticles when acting as signal reporter will increase signal significantly and, hence, reduce or eliminate the time to increase target cells to detectable level. Apart from high sensitivity and speediness, nanotechnology-based systems are developed to have reasonable reproducibility, cost-effectiveness, robustness, and user-friendly properties, allowing their applications even in field applications. The techniques with nanoparticles require universal testing instrument available in most laboratories such as spectrophotometer, fluorescent microscope, and luminometer and some reactions could be observed with naked eyes.

**Figure 1:**
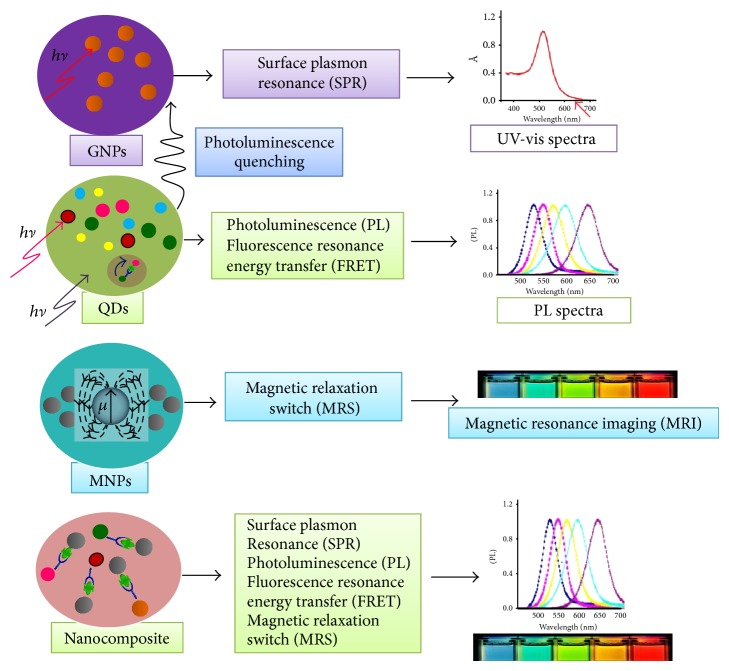
Schematic diagram illustrating different nanoparticles conferring optical (e.g., gold nanoparticles (GNPs)), fluorescence (e.g., quantum dots (QDs)), and magnetic (e.g., magnetic nanoparticles (MNPs)) properties, and combinations between these particles as nanocomposites conferring multifunctionalities provide distinct advantages for environmental monitoring.

Moreover, assays without any sample preparation have been established using innovative nanotechnological tools, leading to user-friendly platforms with rapid and reliable results [19]. As presented in Figure 1, different nanoparticles show specific optical, fluorescence, and magnetic properties, and integrations between these properties hold great promise for environmental screening. In particular, the applications of nanoparticle-based technology enable us to monitor or even improve quality of air, water, and soil. For example, silica nanoparticles are considered an appropriate choice with multiple functional abilities as to deliver antimicrobial agents for treating towards particular pathogenic microorganisms and to sense the microorganisms [20]. Therefore, this further section summarizes the impact of sensing nanotechnology on improving the current testing procedures for accurate and precise monitoring of environmental pathogens and other contaminants.

## 3. Necessary Characteristics for Development of Nanoparticle-Based Biosensor

**Figure 2:**
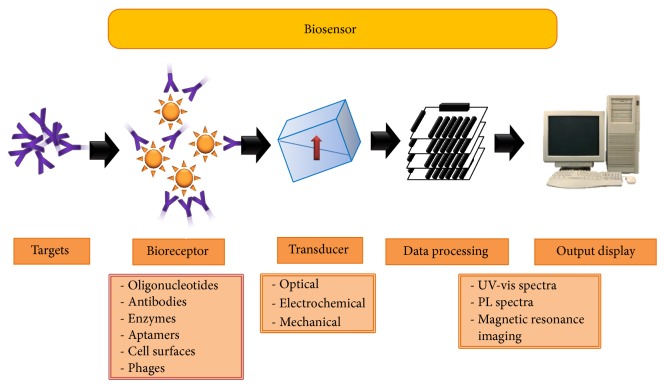
Scheme representing nanobiosensor components consisting of different bioreceptors (e.g., antibodies, aptamers, cell-surface molecules, enzymes, oligonucleotide probes, and phages) and major transducers depending on types of signal response (i.e., optical, electrochemical, and mechanical signal). Output can be displayed as UV-visible or photoluminescence spectra and magnetic resonance images.

Biosensor is typically comprised of two main components: a bioreceptor and a transducer [21]. The bioreceptor is a biomolecule that recognizes the target analyte whereas the transducer converts the recognition event into a measurable signal. The bioreceptor is a biological molecular species (e.g., antibody, enzyme, and nucleic acid), a living biological system (e.g., cells, tissue, or whole organisms), or biomimetic material (e.g., synthetic bioreceptor) that utilizes a biochemical mechanism for recognition. The transducer is a device capable of converting a signal in one form to another form of energy. For transducer classification, common techniques include optical (e.g., luminescence and absorption), electrochemical (e.g., current and voltage), and mechanical measurements (e.g., magnetic resonance). In principle, the detection occurred by the specific binding of target analyte to the complementary biorecognition element (namely, bioreceptor) immobilized on an appropriate supportive medium. The specific interaction causes alteration in one or more physicochemical properties that is detectable using the second component, so-called transducer. Usually, magnitude of signal is proportionally related to the concentration of a specific analyte captured by the biorecognition element [22] (Figure 2).

For development of a biosensor system, some requirements for commercialization are as follows [23].

- *Specificity*. The biosensor device should be highly specific to the target analyte and exhibits minimum or no cross reactivity with moieties conferring similar chemical structure.
- *Sensitivity*. The biosensor device should be able to measure in the range of a given target analyte of interest with minimum additional steps such as precleaning and preconcentration of the samples.
- *Response Linearity*. The linear response range of the system should cover the concentration range over which the target analyte is measurable.
- *Reproducibility*. When samples at same concentrations are analyzed several times, they should produce same signal intensity or magnitude.
- *Short Response and Recovery Time*. The biosensor device response should be rapid enough for real-time monitoring of the target analyte. The recovery time of the biosensor system should be short enough for efficient reusability.
- *Stability and Operating Life*. The signal of biosensor device response should be stable enough for real-time monitoring of the target analyte. The components of biosensor device should be resistant to deterioration throughout the operating period. The operating lifetime should be long enough for monitoring of the target analyte. Of concern, most of the biological components are unstable in different biochemical conditions.

## 4. Characteristics of Environmental Nanobiosensors Based on Potential Nanomaterials

**Figure 3:**
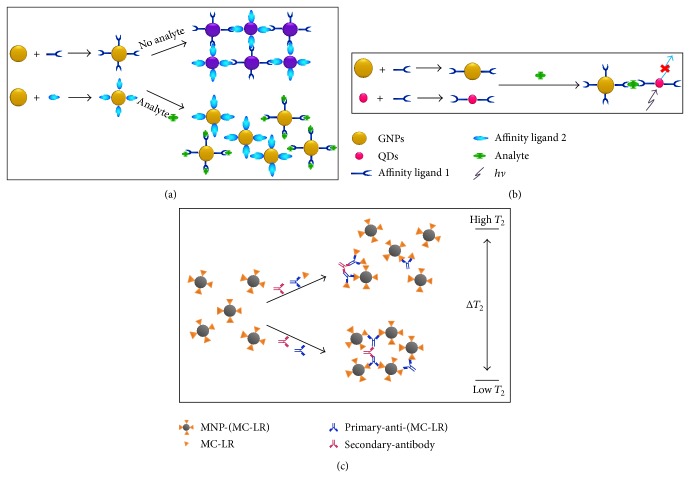
Scheme depicting principle of biosensor-based detection using (a) gold nanoparticles and (b) quantum dots as well as (c) magnetic nanoparticle aggregates for detection of microcystin-LR (MC-LR), naturally occurring toxin produced from cyanobacteria.

Biosensor is defined as a device or an assay with use of a biorecognition element coupled to a signal transducer for measurement an analyte of interest [24]. Common biorecognition elements include oligonucleotide probes, antibodies, enzymes, aptamers, cell-surface molecules [25], and phages [26]. Transducers are divided into three main types: optical, electrochemical, and mechanical. As shown in Figure 3, schemes illustrate fundamental principle of biosensor-based detection: the full spectrum of biorecognition elements and transduction methods was reportedly established for detection of particular water-borne pathogens, with oligonucleotide probes and antibodies being the most common.

### 4.1. Nanoparticle-Based Optical Sensors

Gold nanoparticles (GNPs) that are widely used have various nanostructures. Owing to their low dimensionality and relevant properties, they are thus introduced into novel applications in photonic, electronic, and sensing sections. With color and fluorescence properties of gold nanoparticles and quantum dots, they are commonly utilized in optical sensors for detection of toxins, heavy metals, and other environmental contaminants, as discussed below. Typically, gold nanoparticles display various colors (ranging from red to purple or blue) depending on interparticle reactions during aggregation or dispersion of the aggregates (Figure 3(b)) [27] while quantum dots exhibit changes in photoluminescence intensity (Figure 3(b)) [27]. Optical sensors are served as powerful tools for detecting environmental contaminants since they exhibit high signal-to-noise ratios.

Potential natural toxins, such as ochratoxin A (OTA), zearalenone (ZEA), and aflatoxin B1 (AFB1), are produced from certain fungi* Aspergillus ochraceus, Aspergillus flavus, Aspergillus parasiticus*, and* Fusarium graminearum*. They are often contaminated in cereals, cereal products, and coffee beans and exhibit teratogenic, mutagenic, and immunosuppressive activity in human. Moreover, another class of toxins is also generated from particular bacteria* Bacillus botulinus*,* Escherichia coli*, or* Ricinus communis* that are usually found in animal tissues and plant. These toxins can produce high toxicity and induce injury to human. Owing to high affinity of the toxin ricin to sugar underlying the naturally occurring infection mechanism, gold nanoparticles were coated with sugar to detect ricin and readout can be visualized [28]. Combination of chromatographic technology with conventional immunoassays (namely, immune-chromatographic strip assays) facilitates a rapid and affordable tool for toxin diagnosis [29]. Different types of immune-chromatographic assays with use of gold nanoparticles-antibody conjugates were developed for detection of ochratoxin A [29, 30], zearalenone [31], and aflatoxin B1 [32]. The immune-chromatographic strip assay has advantages in terms of format simplicity, speediness, and stability over a wide range of conditions, allowing for on-site testing by untrained users.

Of ongoing global concern, heavy metal contamination in environment can cause problems on both public human and environmental health. Hence, environmental monitoring of aqueous heavy metal ions becomes crucial. A variety of nanoparticles-based sensors for sensing selective heavy metal ions have been successfully conducted. By using hyper-Rayleigh scattering technique, a gold nanoparticle-based sensor was utilized for rapid screening of mercury (Hg^2+^) ions in aqueous solutions with a sensitivity of 5 ng/mL (ppb) [33]. Additionally, L-cysteine-functionalized gold nanoparticles were employed to detect aqueous copper (Cu^2+^) by changing solution color from red into blue [34]. This colorimetric nanosensor enables rapid, quantitative detection of Cu^2+^ with a sensitivity of 10^−5^ M. Likewise, based on mediated T-T base pairs at room temperature, a novel and practical colorimetric detection of Hg^2+^ was developed using 14 nm nanoparticles with sensitivity as low as 3.0 ppb of Hg^2+^ by unaided eye [35].

Numbers of optical sensors have been continuously established based on photoluminescent-quenching characteristics. A gold nanoparticle-rhodamine 6G-based fluorescent sensor was developed for sensing Hg^2+^ in aqueous solution with a detection limit of 0.012 ppb [36]. Similarly, photoluminescence-based assays were performed for monitoring level of Hg^2+^ (with sensitivity of 2.0 ppb) by the fact that Hg^2+^ concentration is directly proportional to photoluminescent intensity [37]. In addition, a homogeneous Cu^2+^ sensing assay was conducted based on photoluminescent-quenching between a perylene bisimide chromophore and gold nanoparticles in the presence of Cu^2+^ [38].

G-quadruplex-based DNAzymes, nucleic acid enzymes with peroxidase-like activity, were utilized for colorimetric and chemiluminescent detection of various metal ions in aqueous samples. In principle that Hg^2+^-induced T-T base pair can stimulate appropriate folding of G-quadruplex DNAs but inhibit the DNAzyme activity, G-quadruplex-based DNAzymes were conducted for detecting aqueous Hg^2+^ with detection limit of 50 nM (10 ppb) [39].

A multiplex assay for detecting Hg^2+^ and silver (Ag^+^) ions was developed using an electron-transfer-quenching path [40]. Hg^2+^ or Ag^+^ ions could modify quantum dots by inducing formation of T-T and C-C base pairs, respectively, resulting in colorless complexes of Hg^2+^-thymine (T) or Ag^+^-cytosine (C) that cannot transfer energy from the quantum dots. Consequently, concentration of Hg^2+^ or Ag^+^ ions is inversely proportional to photoluminescence intensity via electron-transfer quenching. Nevertheless, this assay was not sensitive enough for sensing such Hg^2+^ or Ag^+^ ions. A more sensitive assay was thus developed for highly selective detection of Hg^2+^ ions (with detection limit of 5.0 nM), based on aggregation-mediated fluorescence quenching of 11-mercaptoundecanoic acid- (11-MUA-) protected gold nanoparticles in presence of 2,6-pyridinedicarboxylic acid [41]. The aggregation mainly occurred by interaction of Hg^2+^ ions with carboxylate groups on 11-MUA-protected gold nanoparticles [40].

Furthermore, nanoparticles can be effectively employed to detect small molecules (e.g., hydrogen, carbon dioxide, nitrogen oxide, oxygen, and ammonium ions). Contamination with nitrite (NO_2_) ions from chemical fertilizers, livestock, and organic waste becomes environmental problem. The* in situ* precipitation of gold nanoparticles, a sensitive colorimetric assay for selective detection of nitrite and nitrate contaminants, was developed using gold nanoparticle probe functionalized with nitrite-reactive groups [42]. The 2,4,6-trinitrotoluene (TNT) compound is commonly used as nitroaromatic explosives for mining-related purposes and consequently contaminated into soil and ground water. This TNT compound can be detected by reaction between TNT and cysteine on the gold nanoparticle surface using so-called cysteine-modified gold nanoparticle-based surface enhanced Raman spectroscopy probe in label-free system [43]. In presence of TNT, a gold nanoparticle color becomes altered with a detection limit of 2 pM TNT in aqueous solution.

A fluorescence nanoparticles-based assay for rapid and selective nitrite detection was constituted which relied on nitrite-induced fluorescence quenching of the nanoparticles through a simple diazotization reaction [44]. Under optimal conditions, nitrite was quantitatively determined using organic fluorescence nanoparticles (namely, 1-aminopyrene nanoparticles) under linearity range of 20–1400 ng/mL with a correlation coefficient of 0.9987 and detection limit of 3 ng/mL nitrite in solution. For quantitative determination, this method for nitrite analysis can be applied to water samples.

In addition, gold nanorods can be incorporated into optical sensors for detecting bacterial pathogens. Coliform bacteria (e.g.,* Escherichia coli*) contaminated in the environment is still a serious public health concern. Of necessity, a sensitive assay based on an antibody-conjugated gold nanorod was successfully constituted by using two-photon scattering technique for determining* E. coli*, with detection limit as low as 50 colony forming unit (CFU)/mL [45]. Using gold nanorod assemblies with basic side-by-side and end-to-end modalities, a rapid and sensitive detection method for microcystin-LR (MC-LR) containing two substitutions of leucine (Leu) and arginine (Arg) was further developed, with detection limit of 0.45 ng/mL and 5 pg/mL, respectively [46]. The assemblies with different geometries of MC-LR were determined using adsorption spectroscopy and light scattering. Besides MC-LR, the measurable immunoassembly methods can be extensively utilized for detection of other various environmental toxins.

### 4.2. Nanoparticle-Based Electrochemical Sensors

Electrochemical sensors are of interest to sensor-focused research field. Several enzyme-based systems, similar to glucose sensor, were developed. Nanoparticle-based labels for analyte not only are useful for spectroscopic methods but are also applied in electrochemical detection. Since metal nanoparticles can be oxidized to form ions that are electrochemically detectable, electrochemical sensors thus were often utilized for screening environmental contaminants.

An electrochemical sensor for copper (Cu^2+^) ions was accomplished with detection limit of less than 1 pM [47]. Electrodes were initially established with gold nanoparticles, and then the gold colloid surface was subsequently functionalized with cysteine for sensing Cu^2+^ ions. Single-walled carbon nanotubes (SWNTs) impregnating porous fibrous materials (e.g., fabrics and papers) were employed to render biosensors high performance [48]. SWNTs and antibodies were utilized to create paper-based sensors for sensitive and specific detection of MC-LR. A paper-based sensor was successfully employed to detect microcystin-LR (MC-LR) in Tai lake sample, with detection limit of 0.6 ppb and at least 28 times quicker response period in comparison to that obtained by an enzyme-linked immunosorbent assay [49]. This nanoparticle-based electrochemical sensing technology facilitates the preparation of several other sensitive environmental sensors. Additionally, a sensitive electrochemical immunosensor using analyte-functionalized single-walled carbon nanohorns was developed for detecting MC-LR in Tai lake water [50]. In competitive immunoassay format, the immunosensor using horseradish peroxidase-conjugated MC-LR antibodies showed broad spectrum response of linearity (0.05–20 *μ*g/mL) with detection limit of 0.03 *μ*g/mL. Such nanoparticle-based electrochemical sensing technology would improve prominent tool performance for detecting various pathogens and their potential toxins as well as for on-site monitoring of environmental pollutants.

### 4.3. Magnetic-Relaxation Sensors

Magnetic-relaxation sensors have been established based on the switching events between target analyte-induced aggregation and disaggregation of magnetic nanoparticles (MNPs). Biocompatible magnetic nanoparticles can serve as magnetic-relaxation switches (MRS) by generating spin-spin relaxation times of water T_2_ signals and by resulting switches between dispersed and aggregated forms. Magnetic-relaxation switches-based methods evolve radiofrequency, hence being indifferent to light-based interference (e.g., scattering, absorption, or fluorescence) in fluids or tissues. Magnetic-relaxation switches-based sensing technology is used for detecting analytes, especially environmental toxins in various matrices. In relevance to radiofrequency, magnetic-relaxation switches-based assays enable sensing such complex and nonoptical matrices (e.g., multicomponent environmental samples, blood, or culture media). Consequently, the handling capability with complex samples expedites multiple processing steps, relative to the traditional optical applications [51–54].

In addition, magnetic-relaxation switches-based methodologies provide advantages over similar detection tests* in vitro*. Specific and highly sensitive assays with the use of MRS sensors were able to quantitatively determine bacterial pathogens in environmental samples [55]. For sensing MC-LR residual, stable and sensitive immunosensors were successfully developed on basis of relaxation of magnetic nanoparticles [56]. By using antigen MC-LR conjugated magnetic nanoparticles, MC-LR specific antibodies can aggregate them into clustered forms in liquid media (Figure 3) [27]. In water sample, the MC-LR was quantitatively determined at range of 1–18 ppb with detection limit of 0.6 ppb. Due to advantage of magnetic-relaxation switches-based assay, it is regarded as a potential platform for rapid monitoring of hazardous pollutants in complex environmental samples and may extend its use of choices in wider fields [8].

## 5. Improvements for Environmental Nanobiosensors Regarding Bioreceptors

**Figure 4:**
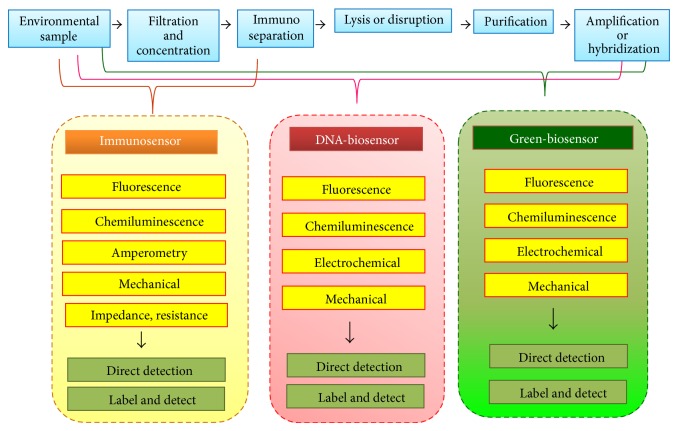
Common procedures for detection of certain water-borne pathogens in environmental matrices and progressive development of respective bionanosensors including immunosensor, DNA-based sensor, and others. Irrespectively, preprocessing steps of necessity initially require filtration and concentration, and then an immunoseparation step (e.g., immunomagnetic separation) in several types of assays.

Recently, nucleic acid biosensor-based researches have been increasingly focused. Nucleic acid biosensors offer desirable sensitivity for detecting particularly water-borne pathogens even at low levels (Figure 4) [57, 58]. At diverging point, immunosensors (left panels) require only filtration, concentration, and detection while DNA biosensors (central panels) possibly need consecutive preprocessing of cellular component disruption, genetic material purification, and often enzymatic amplification and/or hybridization. Aforementioned sensors can be established in either label-based or label-free detection system. Typical detection methods are exemplified in column boxes.

To improve efficacy of these biosensors, step of purification and concentration of pathogens of interest, followed by lysis step of several components (e.g., cell membrane, oocyst wall, spore coat, or viral capsid) and nucleic acid purification prior to amplification and detection, are required. Although market ready-to-use kits for these procedures (e.g., Qiagen's nucleic acid purification kits) are available, such preprocessing steps consume time. The assay procedures need to be simplified and shortened.

In respect to miniaturization, confining reaction within a micro- or nanoscale, fluidic panel has potential to shorten assay time using its higher diffusion ability [59] and to simplify assay steps by combining multiple operations together into micro total analysis systems (*μ*TAS) [60]. Microfluidic devices with sample preparation steps including immunoseparation and preconcentration have been accomplished for mRNA isolation [61], PCR-based amplification [62–65], and isothermal amplification reactions [66].

Of particular interest, immunosensors-based methods have proved to enhance sensitivity. Similar to PCR-based methods, these methods possess low detection limits toward detection of numerous pathogens but amplification step is unnecessary. This technology is promising due to the reduction of both assay time and complexity. For instance, biobarcode assays have been employed to accomplish signal amplification in* S. enterica *Enteritidis-sensing assays. A sandwich hybridization assay combining target-specific probe coated-gold nanoparticles and fluorescein-labeled barcode DNA (a 1 : 100 ratio) with magnetic beads was achieved to detect 0.25 fmol target DNA [67]. The detection limit of this method is similar to that of using liposomal signal amplification in DNA-based sensors [68, 69]. Nevertheless, its sensitivity is insufficient to detect nonamplified target DNA. The use of multiple liposome-tagged probes in a rapid lateral flow assay was capable of selectively detecting 16S rRNA as a potential target as 80% of the total RNA (135 ng) in an intact bacterial cell, without enzymatic amplification within 20 minutes [70, 71]. This implies further potential utilization of liposome-based signal amplification.

Another method with use of up-converting phosphor technology (UPT) provides specific signals in assays without enzymatic amplification. Usually, UPT uses inorganic microcrystals that confer visible light emission when an infrared laser is exited, yielding specific signals with very low noise due to nonautofluorescent property [72]. PCR-based assays using these UPT-based molecules as reporters were achieved to detect low level of specific target DNA [73]. Moreover, amplification-free hybridization-based DNA assay using four probes (two labeled with biotin for capture of the specific target and two labeled with digoxigenin) and UPT-reporters labeled with an antidigoxigenin antibody was developed to detect* Streptococcus pneumonia* [74]. With the utility of the multiple probes, this assay enables the detection of target genomic DNA at 1 ng or about 10^6^ bacterial cells.

## 6. Future Perspectives with Advanced Nanotechnology

Of considerable interest, we summarize recent progress in environmental sensor-based research with “individual or combinatorial” uses of fluorescent nanoparticles and magnetic nanomaterials as environmental monitoring tool, and the utility of newly developed nanoparticles for detection of various environmental pollutants [75].

Due to facile synthetic processes of nanoparticles with desirable sizes and structures, this will definitely facilitate development of nanomaterial fabrication. Accordingly, nanocomposites comprising of discrete domains of different materials display novel physicochemical properties that will be important for wider applications in several fields, including environment. For instance, magnetic Fe_3_O_4_ nanoparticles with silica shell in Fe_3_O_4_/SiO_2_ core-shell structures were synthesized. The Fe_3_O_4_/SiO_2_ core-shell absorbed with gold nanoparticles by electrostatic adsorption to amino groups on the surface was established to form Fe_3_O_4_/SiO_2_/Au structures [76]. Composite core-shell nanostructures possessing optical, magnetic, catalytic, and surface plasmon resonance properties offer advantages over individual single-component materials.

Typically, environmental screening is constituted using instrumental analysis (e.g., thin layer chromatography (TLC) [77], high-pressure liquid chromatography (HPLC) [78, 79], gas chromatography-mass spectrometry (GC-MS) [80], liquid chromatography-mass spectrometry (LC-MS), and immunoassay [81–84]). TLC is a simple and economic method for environmental contaminant determination but its sensitivity is low. Traditional instrument-based methods (e.g., HPLC, GC-MS, and LC-MS) are commonly applied for environmental pollutant measurement. However, they are cost- and time-ineffective due to complicated sample preparation [85]. Hence, these methods are inappropriate for routine monitoring of numerous samples. Nanoparticle-based sensors conferring sensitive and specific potential in possible portable platform offer advantages over traditional instrument analysis and enzyme-linked immunosorbent assay in more rapid results and higher throughputs.

Applications of nanoparticle-based sensors in widespread surveillance of environmental toxicants+ are due to their sensitivity, selectivity, speediness, and affordability. The detection of environmental pollutants with fewer steps is possible with nanoparticle-based sensors (e.g., optical and magnetic resonance sensors). Numerous nanosensors have been developed as portable devices. In addition, immune-chromatographic strip-based assays can be readout by unaided eyes. However, quantitative analysis of analytes usually needs an array scanner or similar instrumentation. Portable strip readers for quantitative dry-reagent strip sensors have been designed to assess the color intensity of membrane bands, enabling them for on-site detection [86–88]. On basis of magnetic nanomaterials, magnetic-relaxation sensing method can be undertaken using miniaturized diagnostic magnetic resonance systems containing planar microcoils, microfluidic channels, and a portable magnet [89]. This portable device has been established for rapid, quantitative, and multiplex measurement of multicomponent environmental samples with high sensitivity using smaller device [8].

In commercial setting, these technologies include immunomagnetic separation (IMS) with semiautomated procedure (TCS Biosciences Isolate System), immunofluorescence assay (FA) microscopy in antibodies-labeled well slide platform (Meridian Biosciences MERIFLUOR), cytometry using fluorescent cell labeling and laser scanning technology (highly-automated, ChemScan RDI Solid-Phase Cytometry, bioMérieux), and molecular biology- and PCR-based detection methods of target specific sequences (RT-PCR Detection Kits, CEERAM, and Norgen Biotek).

Advanced nanotechnology refers to the study of how nanotechnology can benefit the environment and hence aims for products and processes that are safe and energy efficient, reduce waste, and lessen greenhouse gas emissions. The so-called “green” nanotechnology is also about manufacturing processes that are economically and environmentally sustainable. Green nanotechnology is increasingly referred to in connection with other concepts such as green chemistry and sustainable and green engineering and manufacturing. This green nanotechnology enables advance development of nanotechnology to minimize potential environmental and human health risks associated with the manufacture and use of engineered-nanomaterial products and to encourage replacement of existing products with novel nanomaterial-based products that are more eco-friendly throughout their lifecycle.

Intriguingly, selective colorimetric assay with the use of green synthesized silver nanoparticles from plant extracts has been recently developed for sensing toxic metal ions in aqueous solution across a wide pH range (2.0–11.0) [90]. The green silver nanoparticles were synthesized by coordinating metal with organic functional groups present in the plant extracts (e.g., fresh and sun-dried neem leaf, fresh and sun-dried mango leaf, green tea, and pepper seed). Fresh neem leaf extracts-based silver nanoparticles were selectively capable of detecting Hg^2+^ while sun-dried neem leaf extract-based silver nanoparticles were found to selectively determine Hg^2+^ and Pb^2+^ at micromolar concentrations. Neem bark extract-based silver nanoparticles displayed selective colorimetric sensing of Hg^2+^ and Zn^2+^. Similarly, silver nanoparticles synthesized from mango leaf (fresh and sun-dried) and green tea extracts exhibited selective colorimetric sensing of Hg^2+^ and Pb^2+^ ions. Interestingly, pepper seed extracts-based silver nanoparticles showed selective colorimetric sensing properties toward Hg^2+^, Pb^2+^, and Zn^2+^. These green synthesized silver nanoparticles offer versatility with use of plant extracts via green nanotechnology as well as applicability in environmental sensor, especially in decontamination of toxic metal ions over broad pH range.

Taken together, high-throughput and ultrasensitive detection nanotechnology provide effective screening methods for various environmental pollutants. Nanosensors offer potential advantages: sensing capability of microbial pathogens or chemical contaminants at very low levels, convenient handling as portable device for on-site screening or real-time monitoring, cost-, labor-, and time-effectiveness, and simultaneous multiplex detecting ability. Furthermore, advanced nanotechnology encourages a fresh way of designing new products, with the environment and sustainability in mind. This nanobiotechnology will further promote widespread applications in several fields, particularly in environmental monitoring. The benefits of nanotechnology have thus an important role in keeping the environmental health safer.

## 7. Concluding Remarks

Of global health concern, particular water-borne pathogens and other potential toxicants contaminated in environmental conditions are critical. Development of detection methods with sensitivity, selectivity, and speediness is urgently required for screening their occurrence in correspondence with safety regulations at clinically significant levels. This will promote for betterment of the public health and individual life quality. Even though nucleic acid-based biosensors have potential at sensing very low concentrations, they still require time-ineffective purification steps at upstream processes. Immunosensors need relatively fewer steps of sample preparation processes, giving rise to shorter assay time; however, antibodies of need are complicated and noneconomical. Using different signal amplification and background-reduction techniques coupled with the miniaturization with enhanced sensitivity, nucleic acid/antibody-based detection methods offer sensitive and selective tools for screening various forms of water-borne pathogens.

So far, current investigations have been focused on detection of pathogens in the actual environmental samples as well as on prerequisite of preprocessing steps. Combinatorial use of fluorescent nanoparticles and magnetic nanomaterials will facilitate miniaturization techniques, multiplex detection systems, and nanomaterials-based research for simultaneously sensing relevant pathogens in a specific environmental scenario. However, some artifacts relating to interfering substances, nonspecific binding, aggregation, and toxicity of such nanoparticles should be addressed prior to their full potential and implementation as biosensors. The significant advantage includes rapid results because the approach to increase signal rather than the target analytes has revolutionized the paradigm of detection.

Taken together, these methodologies conjugated with green nanotechnology will expedite potential existing methods that would offer sensitivity, specificity, speediness, robustness, and self-cleaning to complement or replace the typical standards as well as promote accessibility of safe drinking water and decrease the global health problem due to water-borne diseases, in particular. Taking care of environmental concerns up front pays back in long-term benefits.
